# Proliferative Kidney Disease and Proliferative Darkening Syndrome are Linked with Brown Trout (*Salmo trutta fario*) Mortalities in the Pre-Alpine Isar River

**DOI:** 10.3390/pathogens8040177

**Published:** 2019-10-06

**Authors:** Daniela Arndt, Robert Fux, Andreas Blutke, Julia Schwaiger, Mansour El-Matbouli, Gerd Sutter, Martin C. Langenmayer

**Affiliations:** 1 Institute for Infectious Diseases and Zoonoses, LMU Munich, 80539 Munich, Germany; daniela.arndt@micro.vetmed.uni-muenchen.de (D.A.); robert.fux@lmu.de (R.F.); martin.langenmayer@gmx.net (M.C.L.); 2 German Center for Infection Research (DZIF), Partner Site Munich, 80539 Munich, Germany; 3 Research Unit Analytical Pathology, Helmholtz Zentrum Munich, 85764 Neuherberg, Germany; andreas.parzefall@helmholtz-muenchen.de; 4 Bavarian Environment Agency, Unit Aquatic Toxicology, 82407 Wielenbach, Germany; julia.schwaiger@lfu.bayern.de; 5 Clinical Division of Fish Medicine, University of Veterinary Medicine, 1210 Vienna, Austria; Mansour.El-Matbouli@vetmeduni.ac.at

**Keywords:** immunohistochemistry, PDS, piscine orthoreovirus 3, PKD, quantitative stereology, *Tetracapsuloides bryosalmonae*

## Abstract

For many years, brown trout (*Salmo trutta fario*) mortalities within the pre-alpine Isar River in Germany were reported by the Bavarian Fisheries Association (Landesfischereiverband Bayern e.V.) and local recreational anglers during August and September. Moribund fish seemed to be affected by proliferative darkening syndrome (PDS). In addition, proliferative kidney disease (PKD) caused by *Tetracapsuloides bryosalmonae* was discussed. To investigate this phenomenon, the present field study monitored brown trout mortalities by daily river inspection in 2017 and 2018. Moribund brown trout (n = 31) were collected and examined using histology, immunohistochemistry, qPCR, and quantitative stereology. Our investigations identified 29 (93.5%) brown trout affected by PKD. Four brown trout (12.9%) displayed combined hepatic and splenic lesions fitting the pathology of PDS. The piscine orthoreovirus 3, suspected as causative agent of PDS, was not detectable in any of the samples. Quantitative stereological analysis of the kidneys revealed a significant increase of the renal tissue volumes with interstitial inflammation and hematopoietic hyperplasia in PKD-affected fish as compared to healthy brown trout. The identified *T. bryosalmonae* strain was classified as part of the North American clade by phylogenetical analysis. This study highlights PKD and PDS as contributing factors to recurrent autumnal brown trout mortalities.

## 1. Introduction

In the last decade, high mortalities and a dramatic decline of the brown trout populations were reported by the Bavarian Fisheries Association (Landesfischereiverband Bayern e.V.) and local recreational anglers in the pre-alpine Isar River in Southern Bavaria (Germany). These mortalities were predominantly observed in the warmer summer months. Facing the advent of global climate change, aquatic ecosystems are particularly endangered because increasing water temperature is an important factor for aquatic pathogen spread and multiplication [1,2]. Autochthonous species, specialized in colonization of specific habitats, are especially vulnerable to changes of their microenvironment [3]. In the Isar River, the decline seems to solely affect brown trout (*Salmo trutta fario*). Thus, adult brown trout, capable of reproduction, were regularly restocked in the past years in order to regenerate and stabilize the natural populations. Still, stocking failed to halt the decline of brown trout. Adult brown trout capable of reproduction were mainly found moribund or already dead during August and September. The scale of the decline is probably high, but precise data is missing because declined brown trout populations are hard to monitor. Anglers described apathetic behavior and black discoloration of moribund brown trout. These rather unspecific symptoms have been described for various different fish diseases [4]. However, these clinical signs also match two disorders affecting brown trout from other rivers in the Alpine region: proliferative darkening syndrome (PDS) and proliferative kidney disease (PKD) [5,6]. So far, none of these diseases have yet been reported to occur in the Isar River and various other pre-alpine rivers in Bavaria.

For decades, PDS has caused a massive decline of brown trout populations in the grayling zone of several pre-alpine rivers in Southern Germany [6,7]. These mass mortalities affect a major part of the brown trout population every late summer until autumn, whereas rainbow trout (*Oncorhynchus mykiss*) populations are not affected. The specific cause of PDS is unknown and potentially multifactorial. Piscine orthoreovirus 3 (PRV-3) has been recently suggested as the potential cause of PDS [8], but this hypothesis could not be confirmed and rather PRV-3 seems to cause a subclinical bystander infection [9]. Despite various investigations within the last decade, a causative agent has not been detected yet [6,8]. Therefore, the diagnosis of PDS is solely based on epidemiological data (seasonal occurrence), macroscopic lesions (darkening of skin), and histological lesions (liver necrosis in combination with depletion of splenic white pulp) [9]. PKD has recently been suggested as a potential cofactor in PDS [10]. The responsible parasite *Tetracapsuloides bryosalmonae* already represents a relevant pathogen for trout in Austrian rivers [10].

PKD is a parasitic disease in farmed and wild rainbow and brown trout with a strong immunosuppressive character and the capacity to cause high mortalities which particularly occur in the northern hemisphere [5,11,12,13]. Besides darkening of the skin, PKD-affected fish mainly show swelling of the kidneys and anemia [14,15], pale yellow livers are also noticed [15].

The causative myxozoan, *Tetracapsuloides bryosalmonae*, has a complex two-host lifecycle within freshwater bryozoa, mostly *Fredericella sultana* [16,17]. Freshwater fish are infected through gills or skin [18,19]. The parasite migrates through blood vessels into its main target organ, the kidney [17,20,21]. In the kidney, the parasite is located within the interstitium and the tubules causing interstitial inflammation and hematopoietic proliferation [5,15]. After renal development of the parasite, spores are produced and released via urine to start a new lifecycle [21,22,23]. In severe courses of the disease, hematopoietic hyperplasia and inflammation in the kidney result in impairment of renal function [15,24]. PKD also leads to immunosuppression by dysregulation of the cellular immunity making the infected fish more susceptible to secondary infections [13,25].

Water temperature plays an important role in the pathology of PKD-infected fish. Clinical signs and mortality are significantly increased above 15 °C [26,27]. Mortality rates in wild fish are usually less than 20%, however in the presence of secondary infections mortality rates are probably higher [5,14,28].

The brown trout decline, partially caused by PDS, is suspected to occur in a large body of pre-alpine rivers and its effect on brown trout populations is probably massive [6]. However, neither presence nor prevalence of various piscine pathogens have been characterized in detail for most southern German rivers. This is also true for the third largest river in Bavaria, the Isar. The apparent brown trout decline warrants qualitative and quantitative investigations to highlight potential causes, to provide a basis for epidemiological investigations and the identification of countermeasures and to improve the knowledge on interrelated disease processes. Thus, the present study was initiated by the Bavarian Fisheries Association (Landesfischereiverband Bayern e.V.), with local anglers reporting annual recurring brown trout mortalities in the Isar River. In this field study, we investigate specimens of the brown trout mortalities in 2017 and 2018 using state-of-the-art pathological and molecular biological analyses. Lesion distribution and quality were pathologically assessed. Parasite burdens and their impact on inflammatory lesions and tissue damage in diseased brown trout were determined with qPCR analyses and unbiased quantitative stereology. Our results demonstrate that PKD and PDS contribute to the recurrent mortalities of Isar brown trout. 

## 2. Results

### 2.1. Brown Trout Mortalities Occur at Specific Isar River Sections within Munich

**Figure 1:**
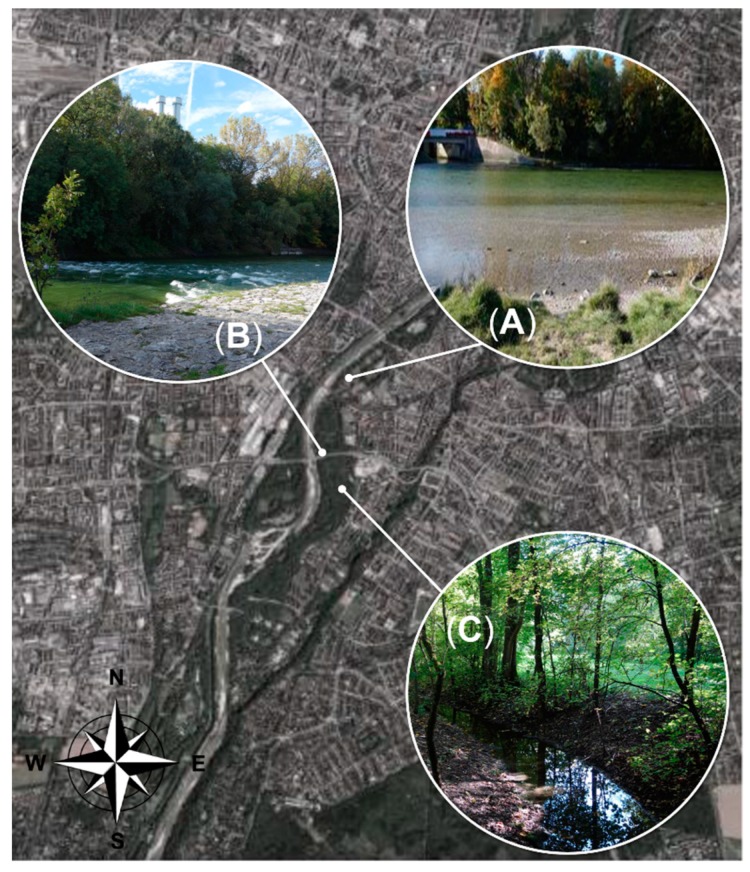
Sampling sites at the Isar River, Munich: (**A**,**B**) 2017 hotspots, main stream of the river (**A**: 48°07′06.7″ N 11°33′40.3″ E, 48°07′10.1″ N 11°33′43.0″ E; **B**: 48°06′44.3″ N 11°33′34.3″ E); (**C**) 2018 hotspot, Isar meadows, Auer Mühlbach (48°06′45.3″ N 11°33′46.5″ E).

In 2017, moribund brown trout were only observed at specific sites in the main stream of the Isar River. In 2018, most fish were observed in the meadows, in a smaller side stream called Auer Mühlbach (Figure 1).

**Figure 2:**
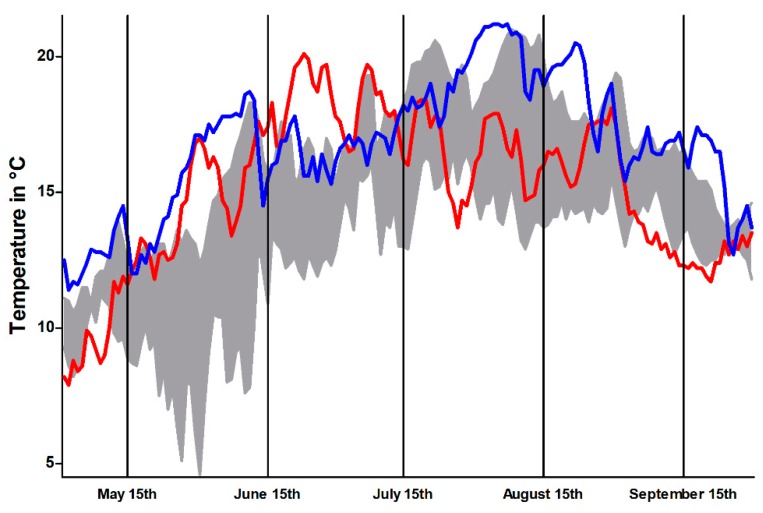
Temperature profile, representing mean daily temperature within the Isar River during sampling periods in 2017 (red) and 2018 (blue), compared with temperature range of the preceding four years (grey area). Data from the Bavarian Agency of Environment (https://www.gkd.bayern.de).

During the observation period, multiple dead but relatively few moribund brown trout were observed in both years. In total, 24 moribund brown trout were sampled in 2017 and seven in 2018. Apparent mortalities in other fish species were not observed. Affected brown trout were apathetic and swimming velocity and general activity were greatly reduced. The fish avoided the water current, stood near the shore, and lost their natural flight reaction. Collected brown trout had a mean size of 35.5 cm (standard deviation [SD] = 4.5) in 2017 and 28.6 cm (SD = 4.6) in 2018. Female and male brown trout were equally affected (19 and 12 animals, respectively). The water temperature during this time period was not unusually high when compared to the preceding years (Figure 2).

### 2.2. Necropsied Brown Trout Mainly Displayed Renal and Hepatic Lesions

**Figure 3:**
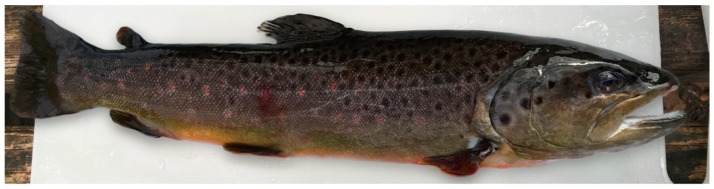
Affected brown trout with black skin color.

All sampled trout underwent pathological examination. The skin of the sampled moribund brown trout was dark and showed a nearly black color (Figure 3). Many brown trout displayed spread opercula before necropsy as a sign of increased respiration. The gills were pale (indicative of anemia) and in one case hemorrhagic. Routinely performed native gill and skin smears were free of microscopically detectable ectoparasites. Exophthalmos was present in 10/31 (32%) of brown trout. One fish showed bilateral corneal opacity. Livers and spleens were mildly enlarged. The anterior and posterior kidneys were swollen with dark red color and a micronodular surface. Body fat stores were considerably reduced, indicating poor nutritional status. 

Bacteriologic culture of heart, liver, spleen, and posterior kidney resulted in bacterial growth in 16/31 (52%) fish. MALDI-TOF mass spectroscopy of these cultures led to the identification of *Aeromonas salmonicida*, the etiological agent of furunculosis, in the posterior kidney of one fish. However, skin and inner organs of this brown trout displayed no macroscopic or histologic lesions compatible with furunculosis. The mass spectra of the other bacteria did not coincide with commonly known fish pathogens and histology did not reveal bacterial infection. Of note, the causative agent of bacterial kidney disease (BKD), *Renibacterium salmoninarum*, could not be identified in cultures from the posterior kidneys and kidney histology did not indicate bacterial infection.

### 2.3. Brown Trout Displayed Lesions Compatible with PKD and PDS

To complement necropsy results, brown trout organs were assessed via histology. Histologic lesions compatible with PDS (defined as combined splenic and hepatic lesions) were detected in 4/31 brown trout (13%). Solitary splenic or hepatic lesions were identified in 27/31 (87%) fish. All individuals with lesions indicating PDS also displayed kidney lesions typical for PKD. In total, kidney lesions compatible with PKD were detected in 29/31 (94%) sampled brown trout.

**Figure 4:**
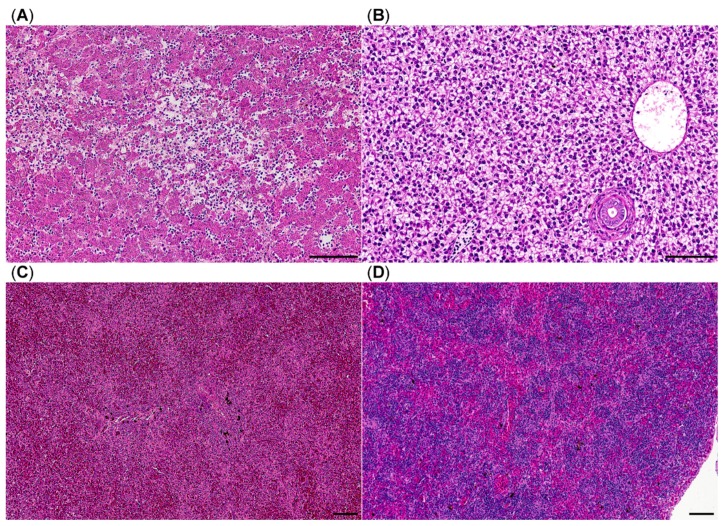
Histology of brown trout liver and spleen, HE stain, (**A**) liver with multifocal liquefactive necrosis (center) and viable hepatocytes without vacuolation suggesting depletion of glycogen stores; (**B**) liver from a healthy brown trout; (**C**) congested spleen with lymphocytic depletion of white pulp areas; (**D**) spleen from a healthy brown trout; bars = 100 µm.

The examined livers showed loss of hepatocyte vacuolation in every specimen suggesting diminished hepatic glycogen storage. Additionally, 7/31 (23%) brown trout displayed multifocal random liquefactive hepatic necrosis (Figure 4). Ten of 31 livers were severely congested and many displayed few perivascular and periportal lymphocytes and histiocytes. In 27/31 (87%) spleens, the lymphocytes of the white pulp were depleted (Figure 4) and red pulp areas congested. Seven of 31 (23%) brown trout displayed gastrointestinal submucosal edema. Liver samples tested by RT-qPCR for PRV-3-specific RNA were negative in all of the brown trout sampled in 2017 and 2018.

**Figure 5:**
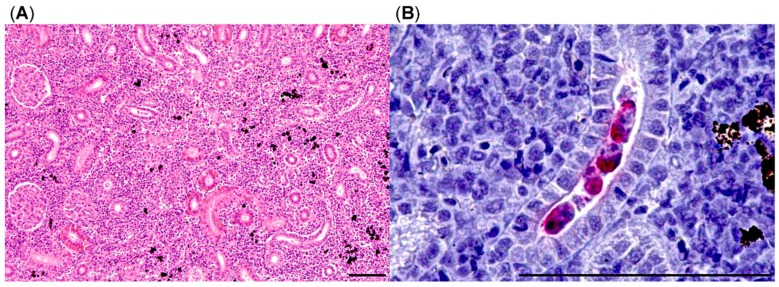
Histology of brown trout posterior kidney, (**A**) increased interstitial cellularity with separation of melanomacrophage centers, HE stain; (**B**) multiple tubular parasitic pseudoplasmodia (in red), anti-*T. bryosalmonae*-immunohistochemistry; bars = 100 µm.

Anterior and posterior kidneys showed mild to moderate interstitial nephritis consisting of an infiltration with lymphocytes and histiocytes. Interstitial hematopoietic tissue was increased as well. The branches of melanomacrophage centers were disrupted due to the increased inflammation and hematopoiesis (Figure 5). Several tubular and interstitial parasitic pseudoplasmodia compatible with *T. bryosalmonae* were detected already in routine stains. To detect intralesional parasites with higher sensitivity, an anti-*T. bryosalmonae*-immunohistochemistry was implemented. In 14/31 (45%) brown trout posterior kidneys interstitial and tubular *T. bryosalmonae* pseudoplasmodia were immunohistochemically detected (Figure 5).

### 2.4. The Majority of Brown Trout Kidneys Were Positive for T. bryosalmonae DNA

**Figure 6:**
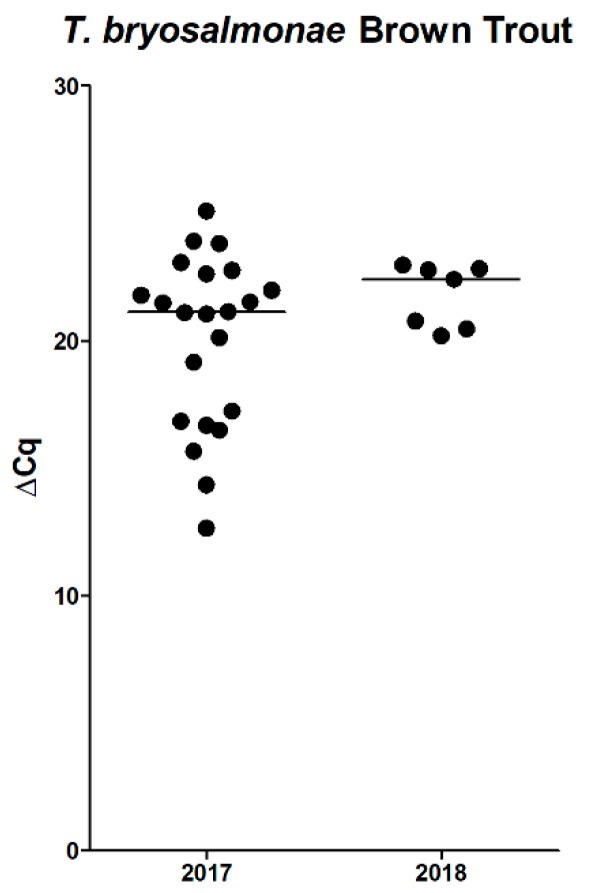
qPCR for *T. bryosalmonae*, of brown trout posterior kidneys, ∆Cq values (2017: 22/24 positive; 2018: 7/7 positive); bars represent medians.

Although immunohistochemistry did confirm *T. bryosalmonae* infection in 14/31 (45%) fish, macroscopic kidney lesions and routine histology suggested a higher rate of infection. Thus, we performed an additional qPCR to detect *T. bryosalmonae* with higher sensitivity and to compare parasite load. In total, 29/31 (94%) kidney samples were determined to be positive by qPCR. High DNA-levels were detected within the kidney of 6/24 brown trout in 2017 with ∆Cq-values over 22, whereas the majority had ∆Cq-values in the range of 15–22. In 2018, all sampled fish showed ∆Cq-values between 20–23 (Figure 6). The alignment with 18S reference sequences (FJ981823, KF731712, KJ150286, KJ150287, KJ150288) proved a 99–100% identity to the *T. bryosalmonae* genome. 

**Figure 7:**
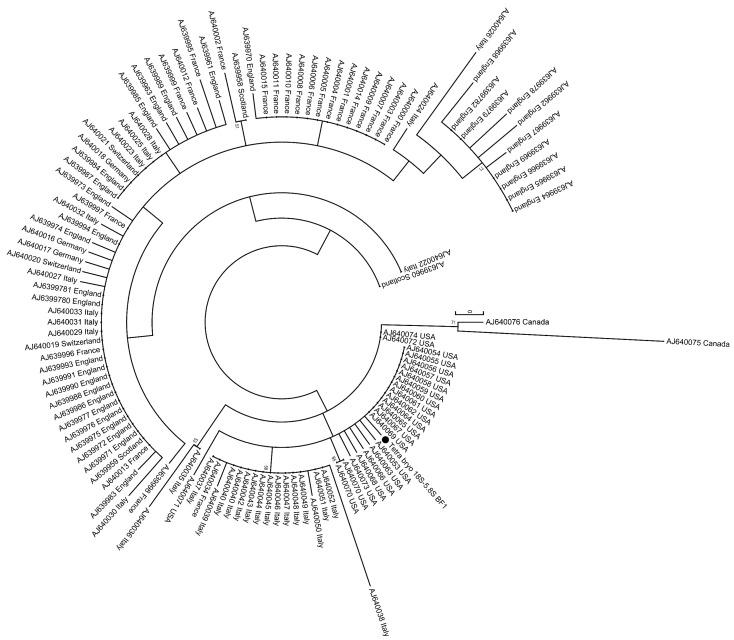
Phylogenetic analysis of *T. bryosalmonae*, including the Isar strain (black dot) grouped into the North American clade of *T. bryosalmonae*.

For phylogenetical analysis we used a genome fragment extending from 18S through the internal transcribed spacer 1 (ITS-1) and terminating in the 5.8S region. The PKD strain found in the Isar River could be assigned to the North American Clade [29] of *T. bryosalmonae* (Figure 7).

**Figure 8:**
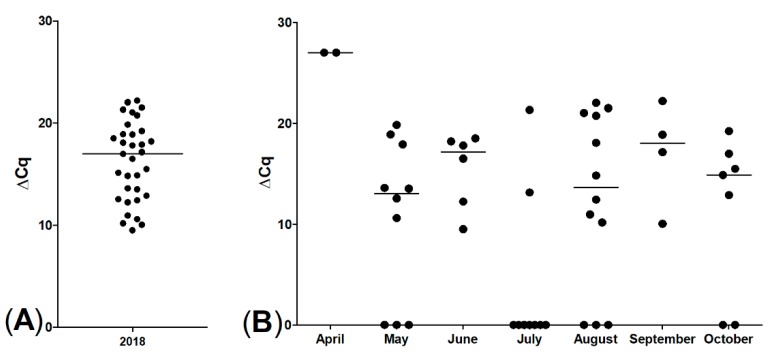
qPCR for *T. bryosalmonae* of rainbow trout posterior kidneys, ∆Cq values, (**A**) 33/50 positive; (**B**) sampling months of all rainbow trout; displaying spread of proliferative kidney disease (PKD) infection during the whole fishing season, bars represent medians.

To monitor PKD presence in healthy trout, we decided to use non-affected brown trout caught by fly fishery before and after reported mortalities occured. During fishing season in 2018 (May–October), selected local anglers were asked to provide kidney samples from caught brown trout. However, neither the authors nor the participating anglers did catch a single brown trout, only rainbow trout were captured (n = 50). Although brown trout were not captured, we still examined the rainbow trout posterior kidneys as a surrogate to assess PKD presence. In the qPCR analysis, 33/50 of rainbow trout posterior kidney samples were PKD positive. The distribution of ∆Cq values of the positive rainbow trout was comparable to the range detected in the brown trout (Figure 8).

In summary, histology and qPCR showed a high prevalence of PKD within all sampled trout. As expected, immunohistochemistry showed a lower sensitivity when compared to qPCR.

### 2.5. Impact of PKD on Renal Inflammation and Tissue Remodelling in Brown Trout

**Figure 9:**
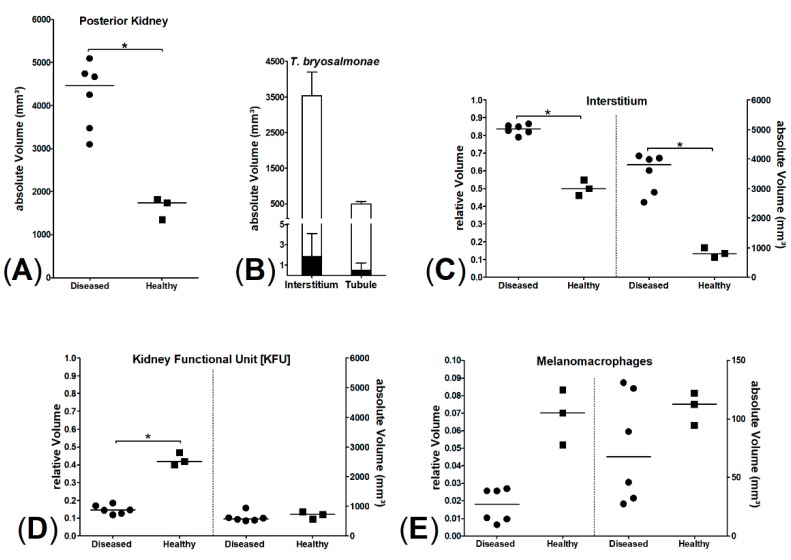
Stereology of posterior kidney of six diseased and three healthy brown trout; significant differences marked by * (*p* = 0.0238, Mann–Whitney test). (**A**) Comparison of posterior kidney volumes, (**B**) parasite volumes (interstitial and tubular parasite stages in sum, black bars) compared to mean interstital and tubular volumes (white bars), (**C**) interstitium, relative (left) and absolute (right) volumes of interstitial kidney tissue, (**D**) kidney functional unit [KFU], relative (left) and absolute (right) volumes of KFU, (**E**) melanomacrophages, relative (left) and absolute (right) volumes of melanomacrophages.

To advance the characterization of the detected kidney lesions, which were associated with the *T. bryosalmonae* infestation, additional quantitative stereological analyses were performed, aiming to objectively quantify the morphological renal changes and connect them to PKD pathogenesis. The median kidney volume of diseased brown trout was significantly higher, as compared to the baseline values of healthy brown trout from the Wielenbach fish hatchery (*p* = 0.0238, Mann–Whitney test; Figure 9A). There was a significant increase of the relative and absolute volumes of interstitial renal tissue (*p* = 0.0238, Mann–Whitney test; Figure 9C). 

The median relative volume of non-interstitial kidney tissue (kidney functional units [KFU]) consisting of glomerular, tubular, and vascular compartments was significantly decreased in diseased brown trout (*p* = 0.0238, Mann–Whitney test). However, the absolute KFU volume was not significantly decreased (*p* > 0.05, Mann–Whitney test; Figure 9D). The increase of total kidney volume in diseased brown trout was thus mainly due to a significant increase in interstitial tissue. Furthermore, the absolute volumes of melanomacrophage centers in the kidney of diseased and healthy brown trout were not significantly different (*p* > 0.05, Mann–Whitney test; Figure 9E). In healthy brown trout, no parasitic pseudoplasmodia could be detected. In diseased fish, parasitic pseudoplasmodia occupied only a minimal amount of kidney volume (Figure 9B), and no statistically significant differences between the absolute volumes of tubular and interstitial parasitic stages were detectable (*p* > 0.05, Mann–Whitney test).

## 3. Discussion

Due to introduction of rainbow trout into German rivers in the early 19th century, autochthonous brown trout populations had to face a strong habitat and food competitor which led to a brown trout decline or even disappearance in many Southern German rivers [30]. With the advent of climate change, the infection pressure on fish increases with rising water temperatures [31]. Two diseases especially devastating for brown trout are influenced by water temperature: PDS and PKD [6,32]. Over the last decades, local anglers have reported a massive decline of the brown trout populations in the grayling zone of several pre-alpine rivers in southern Germany. These mass mortalities were mostly attributed to PDS, because the skin of affected fish often turned dark. In the pre-alpine Isar River, similar observations were made by local anglers and therefore investigations to identify a cause for the decline were initiated.

In this field study we investigated the reported mortalities in the Isar River within the urban area of Munich using state-of-the-art histological, quantitative-stereological, and molecular assays. With these methods we were able to identify and link two major diseases of brown trout, PDS and PKD, to the decline of Isar brown trout. Both conditions can result in rather unspecific skin darkening and are considerably influenced by water temperature [6,11,26]. 

PDS leads to high mortalities in affected brown trout in the summer months [6]. Investigations to identify the cause of PDS have been ongoing for decades, however, up to this date the postulated causative pathogen remains elusive [9]. So far identification of the syndrome relies on histological examination of the affected brown trout. Hepatic necrosis and splenic white pulp depletion with or without gastrointestinal edema are the hallmarks of the disease [6]. In the present study, we chose clinical and histological features to define the presence of PDS: Brown trout had to show skin darkening and combined hepatic necrosis and splenic lymphocytic depletion. Using this definition, four fish were identified showing the same distribution and quality of lesions when compared with the observations in an established controlled PDS-exposure experiment [9]. We are aware, that in our study only few brown trout met the criteria for PDS. This indicates that the definition might be too strict and we miss out some fish with PDS, which only show few/singular lesions. For example, three additional fish displayed hepatic necrosis without splenic lymphocytic depletion. In the aforementioned controlled PDS exposition trials from 2008 and 2009 the extent and coincidence of hepatic and splenic lesions also displayed considerable variations between individual fish affected by PDS [6]. Furthermore, identification of histologic lesions requires a certain threshold of damage, thus making histology a diagnostic tool far less sensitive compared with a nucleic acid detection assay like qPCR. It is tempting to speculate that the true number of PDS-affected brown trout might actually be quite higher than those which met the definition criteria.

To this date, a molecular biologic tool to sensitively detect PDS is not available. Although a piscine orthoreovirus (PRV-3) was recently suggested as the causative agent of PDS [8], a follow-up study could not confirm a direct association between PRV-3 infection and PDS. The qPCR experiments verified viral nucleic acid not only in healthy control brown trout but also failed to detect it in some diseased animals with histologically confirmed PDS [9]. Thus, the potential impact of reovirus infections on the health status of brown trout populations remains unclear. In this study, the PRV-3 specific qPCR on liver samples was negative in all samples.

Like PDS, PKD can also lead to high mortalities, especially in farmed trout [12]. The majority of moribund brown trout in this study were positive for PKD, which is consistent with findings of other studies [33,34]. PKD spreading in wild brown trout populations has been reported before [35,36]. In total, 94% of the brown trout and 66% of the rainbow trout were qPCR positive for PKD. These results suggest an enzootic distribution of its cause *T. bryosalmonae* within the Isar River. Interestingly, positive rainbow trout showed a similar distribution of ∆Cq-values but did not display apparent systemic signs like black skin discoloration or emaciation. The cause of this finding remains speculative; maybe rainbow trout are more robust to environmental changes and can therefore control parasitic infestation.

To accurately estimate the impact of PKD infestation on brown trout health, we implemented quantitative stereological methods in 2018. Clifton-Hadley [15] indices determined in 2017 indicated mild to moderate macroscopic lesions of PKD in brown trout posterior kidneys. However, this semiquantitative scoring is subjective and prone to errors in mildly affected individuals, thus we opted to examine the posterior kidney as PKD target organ using design-based quantitative stereology. This technique encompasses a body of sampling and analysis methods based on the principles of stochastic geometry to receive accurate (i.e., precise and unbiased) estimates of quantitative morphological parameters (such as volume, surface area, or particle numbers) of three-dimensional structures by analysis of two-dimensional physical or optical section planes of these structures. This is done without making assumptions on the size, shape, and orientation of the structures of interest [37,38]. Using appropriate quantitative stereological analysis methods, we were able to demonstrate a highly significant effect of *T. bryosalmonae* infection on the increase of the interstitial tissue compartment of the posterior kidney with unbiased methods when compared to previous studies [15]. Even in kidneys with relatively low Clifton-Hadley scores, the volume of interstitial tissue, i.e., hematopoietic and inflammatory cells, was significantly increased. In PKD-affected trout the degree of renal damage and inflammation is connected to the manifestation of clinical signs in affected fish [15]. 

In addition, the volume of melanomacrophage centers (MMCs) in the posterior kidney was decreased in moribund fish, however, not significantly. MMCs are regarded as immunological kidney guardians and are very plastic immunologic structures. Remodeling of MMCs in fish depends on sex, age, and possibly on occurring infections [39,40]. The findings of this study hint that PKD might have a detrimental effect on MMC volume and possibly MMC function. However, for the detection of a significant effect of PKD on MMC volume, the number of fish in our study was too low.

Recently a link between PDS and PKD has been suggested, with PKD inducing PDS likely via immunosuppression [10]. PKD was also detected in the four brown trout with defined PDS lesions. Although there is some lesion overlap between PKD and PDS, hepatic necrosis is clearly not a distinctive feature of PKD [5,11,41]. To further distinguish PKD from PDS, additional qPCR-tested samples of several brown trout derived from a controlled PDS exposition trial were used to detect PKD infestation [9]. Of these, 2/29 posterior kidney samples from PDS-negative animals were PKD-positive by qPCR. None of the PDS-affected brown trout with combined hepatic and splenic lesions, such as liver necrosis and depletion of the lymphocytic cell population in the spleen, were positive for *T. bryosalmonae* (n = 39, qPCR and histology, data not shown). This finding indicates that PDS can be separated from PKD through characteristic PDS liver and spleen pathology. Furthermore, the two diseases can occur both combined and separately whereby each individual illness can lead to high mortalities within affected brown trout populations.

During the sampling months, the Isar had constantly increased water temperatures. Variations in water temperatures in river systems are not unusual. Increased water temperatures enhance PKD due to positive effects on the development of the parasite and increased severity of pathological lesions [27]. Besides, onset of PDS can be delayed by low water temperature, suggesting that the opposite might also be true and higher water temperatures might enhance PDS [6]. Moreover, a constantly high water temperature likely induces environmental stress for brown trout because they are native to an oxygen rich environment. Long-lasting high water temperatures—as measured during the sampling periods—reduce the oxygen binding capacity and thereby induce stress in affected fish and promote their vulnerability for infectious pathogens [42,43]. In this context, the black discoloration of brown trout—as more or less an unspecific symptom–could have been further aggravated by environmental stress [42]. Thus, similar PKD parasite loads (*vide supra*) in brown trout and rainbow trout might not lead to similar disease effects.

Fish mortalities can also be induced by ecotoxicological events [44]. Although we did not measure water parameters directly, we consider such events unlikely as cause for the observed brown trout mortalities. First, during the last several years, only brown trout were repeatedly observed to be affected over a period of at least two months, which makes a single ecotoxicological event unlikely. Second, no other fish species were affected. Finally, water parameters are regularly analyzed by the Bavarian Agency of Environment which did not communicate ecotoxicological events during the sampling periods. To exclude bacterial causes for the brown trout mortalities we performed bacteriologic culture of several organs. *Renibacterium salmoninarum*, the causative agent of bacterial kidney disease in trout, was not detectable and *Aeromonas salmonicida* could only be cultured from one fish which did not display any lesions of furunculosis. Since histology also failed to reveal lesions caused by bacteria or bacterial colonies, we conclude that the primary cause of the mortalities likely was not a bacterial infection.

In conclusion, PKD and PDS are both linked with the brown trout decline in the pre-alpine Isar River. Characteristic lesions of PKD were observed in several fish and parasite infestation was readily detectable with all applied methods (immunohistochemistry, qPCR, and stereology) and connected to considerable posterior renal alterations. Immunological compromise induced by PKD in combination with environmental stress derived from high water temperature appears likely to further aggravate the vulnerability of brown trout for other infections. As shown within this study, PDS also affects the pre-alpine Isar River. Diagnostics on PDS solely based on histological examinations identified only few brown trout being affected by PDS. As PDS lesions vary and histology is not a very sensitive tool, the real number of PDS-affected brown trout in this study might be considerably higher. Further studies on PDS are needed to identify its causative agent by biomolecular examinations to initiate the development of countermeasures for preservation of autochthonous brown trout populations in pre-alpine rivers. Moreover, the identification of the cause of PDS will enable future experiments to investigate the independence or the link of PDS and PKD.

## 4. Materials and Methods 

### 4.1. Sampling Sites in the Isar River and Fish Specimen Collection

The fish used in this study originated from the Munich city segment of the 292 kilometer-long Isar River including the meadows. This Isar River section underwent exceptional re-naturalization in major efforts to improve and protect the river environment during the last decades. While recognizing the pressures of an urban environment within the sampling area, the removal of concrete or inert constructions in the riverbed and on riverbanks and the replacement with vegetation structures alleviates major damages and allows to restore biodiversity. Typical fish species are known to have functional reproduction and hiding places in this renaturalized habitat and include i.e., Danube salmon (*Hucho hucho*), common barbel (*Barbus barbus*), grayling (*Thymallus thymallus*), and common nase (*Chondrostoma nasus*). The “Die Isarfischer München e.V.”, the tenant of the water body “Isar city”, gave permission for river access in this conservation area. This study was conducted in accordance with national and federal guidelines for animal welfare (German Animal Welfare Act, Tierschutzgesetz; Bavarian Fishery Act, Bayerisches Fischereigesetz). Exclusively adult moribund brown trout were caught and humanely euthanized. No ethical approval was required.

A distance of twelve river kilometers (48°04′28.7″ N 11°32′27.5″ E–48°07′41.1″ N 11°34′49.7″ E) was inspected daily by foot along the river banks to identify and collect adult moribund brown trout, starting at the beginning of August until the end of September in 2017 and 2018. This section of the Isar River was chosen, because dead/moribund brown trout were reported by anglers from this area and accessibility was given. In this river segment, the flat shore zones consisted of mainly gravel substrate. Moribund fish were observed in the main stream (in flat shore zones without strong water current) and in the meadows (Figure 1). Before collection, swimming behavior (velocity, positioning, and flight reflex) and general condition (activity, body color, body condition) were assessed. The fish were caught with a modified fish basket made of thin wire. The basket was round (60 cm in diameter) and both ends were open. Adult moribund brown trout were slowly surrounded with the basket to avoid additionally stress. The basket was pushed down to the ground and fish were carefully caught with a landing net. Fish were euthanized with Tricaine methane sulphonate (1 g/10 L).

After the 2017 sampling period and the initial identification of PKD infection of brown trout, we additionally monitored the Isar River trout population for PKD within the same river segment. Due to local regulations, only fly and spin fishing were allowed to catch trout. Selected fly anglers and two of the authors (DA and GS) caught trout in 2018 by fly fishing. Every angler submitted samples of posterior kidneys from caught trout during May until October 2018. All fish were actively feeding on the fly lure. Anglers reported that the fish showed no obvious signs of organic disease, their nutritional status was good, and their stomachs were well filled. To avoid interobserver variability, trout posterior kidneys were not scored using the in Clifton-Hadley index. However, only rainbow trout were caught during this sampling activity. In these, pathological examination was carried out only infrequently. In total, 50 rainbow trout posterior kidney samples were collected, deep frozen and stored for subsequent examination by qPCR.

All efforts to sample healthy brown trout as control fish from the Isar River failed in 2018 and even discovering and sampling of moribund fish was quite challenging. For baseline values of quantitative stereological kidney analyses, we therefore used three healthy brown trout of the same size as the affected brown trout. These fish were raised and maintained in spring water in the hatchery of the Bavarian Agency of Environment (Wielenbach).

### 4.2. Necropsy, Organ Sampling, and Examination

Every brown trout was necropsied directly after euthanasia and macroscopically examined. A comprehensive organ set sample (brain, gastrointestinal tract, gills, gonads, anterior and posterior kidney, heart, liver, and spleen) for histology was routinely fixed 24–48 h in 4% formaldehyde solution, embedded in paraffin, sectioned and stained with hematoxylin and eosin (HE).

The same organ set was sampled from every brown trout for biomolecular assays (deep frozen −80 °C) from all individuals. 

Bacterial cultures were grown from heart, liver, spleen, and kidney samples using blood and Gassner agar plates. Grown bacterial isolates were picked from agars and specified by matrix-assisted laser desorption/ionization time-of-flight (MALDI-TOF) mass spectroscopy in a Bruker Daltonik MALDI Biotyper. To exclude infection with *Renibacterium salmoninarum*, two brown trout caught in August 2017 in the same river segment were additionally submitted to the Bavarian Health and Food Safety Authority (Erlangen). 

### 4.3. Immunohistochemistry

Tissue sections of anterior and posterior kidneys, liver, and spleen of all 31 brown trout underwent immunohistochemistry to detect *T. bryosalmonae* antigens. Heat-induced epitope retrieval (microwave pressure cooker for 33 min) with a tris buffer at pH 9.0 was used for antigen demasking, followed by avidin biotin blocking and normal goat serum to block unspecific reactions. After determination of the final dilution, a monoclonal mouse antibody (IgG1, P01, Aquatic Diagnostics Ltd, Scotland) was used as the primary antibody (1:50 in tris-buffered saline). To increase sensitivity a biotinylated goat anti-mouse antibody served as the secondary antibody (Vector, BA-9200, Burlingame, CA, USA). After incubation with avidin-coupled alkaline phosphatase (ABC-AP, Vector, AK-5000, Burlingame, CA, USA), Liquid Permanent Red (K0640, Dako Agilent, Glostrup, Denmark) was used as the chromogen. Positive (PKD-infected fish) and negative (substitution of the primary antibody with an irrelevant mouse IgG) controls were included in each assay.

### 4.4. Molecular Biological Investigations

DNA and RNA were isolated from posterior kidney samples using the QIAamp Mini Kit or QIAamp RNeasy Mini Kit, respectively, (Qiagen, Hilden, Germany) according to manufacturer’s instructions. For real time PCR the QuantiTect probe PCR kit or the QuantiTect probe RT-PCR kit were used, respectively. Conventional PCR was performed using the Ready Mix Taq PCR Reaction Mix (Sigma Aldrich, Merck, Darmstadt, Germany).

A TaqMan assay for qPCR was used to detect the 18s RNA gene of *T*. *bryosalmonae* in renal tissue samples. Oligonucleotide primers (F: 5′-TGT CGA TTG GAC ACT GCA TG; R: 5′-ACG TCC GCA AAC TTA CAG CT; 800 nM each primer by Grabner and El-Matbouli [45]) were combined with a newly designed TaqMan probe (5′-FAM-TGG ACA AAC GCA AGC TCC TGA TCT -BHQ1; 400 nM). The thermal profile of the PCR was 95 °C for 15 min, and 42 cycles of 95 °C for 15 s, and 60 °C for 1 min. To verify the correct detection of the *T. bryosalmonae* 18S gene, a highly conserved 435 bp fragment was amplified and sequenced using the primers (F: 5′-CCT ATT CAA TTG AGT AGG AGA; R: 5′-GGA CCT TAC TCG TTT CCG ACC; 500 nM each primer) described by Kent [46]. The amount of parasite DNA is shown with ∆Cq-values, meaning ∆Cq-values = 42-Cq. For phylogenetic analysis of T. *bryosalmonae*, we amplified (F: 5′-GAA TGA CTT AGC GAG AAC TTG GTG GTA; R: 5′ CGC AGC AAG CTG CGT TCT TCA TCG A; 500 nM each primer) and sequenced a 585 bp genome fragment extending from the 18S through the internal transcribed spacer 1 (ITS-1) and terminating in the 5.8S [29]. The PCR products were controlled by agarose gel electrophoresis and sequenced using the PCR primers and the sequencing service of Eurofins Genomics (Ebersberg, Germany). For the alignment and analysis of the obtained sequences, DNASTAR Lasergene and MEGA7 software were used. A clustalW algorithm was used for alignment, and a phylogenetic tree was constructed using the maximum likelihood method (HKY matrix) according to Henderson and Okamura [29].

To exclude an infection with the piscine orthoreovirus 3 (PRV-3), we used a RT-qPCR [9], targeting the S1 segment of the virus in liver samples (F: 5′-ATC TCT GGC ACC ACA AGA TTT; R: 5′-GAC CAT AGC AGG CTT AGC RTT A; 800 nM each primer; probe 5′-FAM-AGA CAG ACC AAY CCK ATG CCC GC-BHQ1; 300nM). To denature viral dsRNA, the eluate was incubated for 10 min at 95 °C before RT-PCR. The thermal profile of the PCR was 50 °C for 30 min, 95 °C for 15 min; and 42 cycles of 95 °C for 15 s, 57 °C for 20 s, and 68 °C for 40 s.

### 4.5. Quantitative Stereological Analyses

The volumes of distinct tissue compartments (i.e., the volumes of functional kidney unit (KFU), of interstitial tissue, of melanomacrophages, and of parasitic pseudoplasmodia) within the posterior kidney were analyzed in six diseased brown trout and three healthy brown trout (raised in spring water), using unbiased quantitative stereological analysis methods. From each fish, five thin transversal slabs (of ~5 mm) of fresh kidney tissue were sampled for PCR investigation, using a systematic uniform random (SUR) sampling design [38]. Subsequently, the kidneys were fixed in situ (whole fish carcasses with removal of all inner organs except the kidneys) by immersion in neutrally buffered 4% formaldehyde solution for 24–48 h. After fixation, the kidneys were carefully excised from the fish carcasses, completely sectioned into equidistant (~5 mm thick), parallel, transversal tissue slabs. [37]. The total kidney volumes (V_kid_) were determined from the section areas of the tissue slabs, using the Cavalieri principle, as described earlier in detail [38,47]. One third of the tissue slabs of each kidney (7–9 slabs/fish) were SUR sampled and embedded in paraffin, maintaining the orientation of their section surfaces. The relative volumes of the different tissue compartments within the kidney (V_V(tissue/kid)_) were determined from the fractional areas of their section profiles and the area of the total renal tissue in HE stained histological sections. The section areas of distinct tissue compartments were determined by point counting [38] in 12–14 SUR selected fields of view (FOV) per slab at 400x magnification. Per case, 10747 ± 1183 points were counted. The total volumes of different tissue compartments (V_(tissue,kid)_) were calculated from their respective volume fractions within the kidney and the total kidney volume (V_(tissue,kid)_ = V_V(tissue/kid)_ × V_kid_).
